# The Immunomodulatory Effects of Selenium: A Journey from the Environment to the Human Immune System

**DOI:** 10.3390/nu16193324

**Published:** 2024-09-30

**Authors:** Rebecka A. Sadler, Bonnie A. Mallard, Umesh K. Shandilya, Mohammed A. Hachemi, Niel A. Karrow

**Affiliations:** 1 Department of Animal Biosciences, University of Guelph, Guelph, ON N1G 2W1, Canada; sadlerr@uoguelph.ca (R.A.S.); ushand@uoguelph.ca (U.K.S.); 2 ImmunoCeutica Inc., Cambridge, ON N1T 1N6, Canada; bmallard@ovc.uoguelph.ca; 3 Department of Pathobiology, University of Guelph, Guelph, ON N1G 2W1, Canada; 4 Adisseo France S.A.S., 10, Place du Général de Gaulle, 92160 Antony, France; amine.hachemi@adisseo.com

**Keywords:** selenium, selenoproteins, immunomodulation, adaptive immune response, innate immune response, immunoglobulin, antioxidant, colostrum, inflammation, microbiome

## Abstract

Selenium (Se) is an essential nutrient that has gained attention for its impact on the human immune system. The purpose of this review is to explore Se’s immunomodulatory properties and to make up-to-date information available so novel therapeutic applications may emerge. People acquire Se through dietary ingestion, supplementation, or nanoparticle applications. These forms of Se can beneficially modulate the immune system by enhancing antioxidant activity, optimizing the innate immune response, improving the adaptive immune response, and promoting healthy gut microbiota. Because of these many actions, Se supplementation can help prevent and treat pathogenic diseases, autoimmune diseases, and cancers. This review will discuss Se as a key micronutrient with versatile applications that supports disease management due to its beneficial immunomodulatory effects. Further research is warranted to determine safe dosing guidelines to avoid toxicity and refine the application of Se in medical treatments.

## 1. Introduction

**Figure 1:**
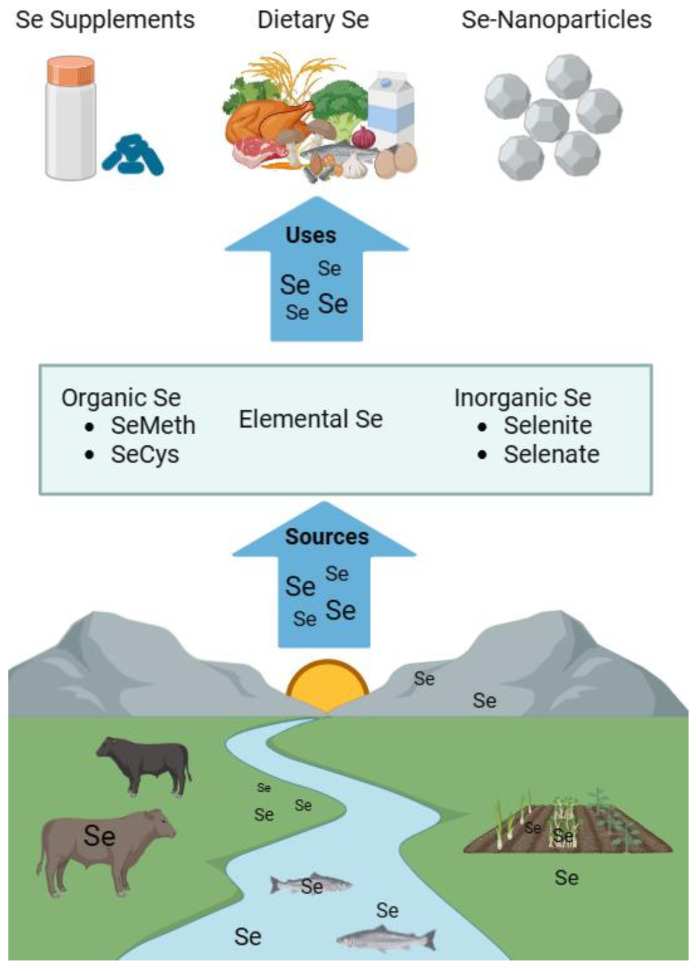
Selenium sources and uses. Se: selenium; SeMeth: selenomethionine; SeCys: selenocysteine [4,5,6].

Several minerals necessary for optimal health are provided by edible plants that accumulate these micronutrients from the environment, namely soils and aquatic sediments. Selenium (Se) is one such beneficial micronutrient. When Se-enriched plants are consumed, the micronutrient is made available to humans and food animals. However, there are certain geographical regions throughout the world where soil Se concentrations are limited or depleted [1]. If diets are restricted to crops harvested from these regions or are otherwise nutritionally unbalanced, Se deficiency may follow. In fact, Se deficiency is estimated to affect approximately 1 billion people worldwide [2]. To overcome this problem, Se can also be provided by supplements (i.e., immunoceuticals [3]) or nanoparticles for medical applications, as depicted in Figure 1.

Among its various health benefits, Se’s role in supporting optimal immune system function is paramount [7]. Our global population is growing rapidly with new diseases increasingly arising, and healthcare systems are being heavily challenged. Therefore, immunomodulatory research is more important than ever so we can fine-tune the acuity of the immune system to help combat existing and novel pathogens in our ever-changing world. Thus, the purpose of this review is to draw on scientific studies using both humans and animal models to explore how Se modulates the immune system, providing updates on the recent advancements in this research area. This review is distinct from others due to its up-to-date and broad inclusion of all forms of Se administration and research drawn from various animal models. Moreover, this review uniquely emphasizes Se’s modulation of the immune system and the broad range of diseases and relevant pathogens Se may help to combat, thus giving the reader a comprehensive overview of Se’s immunomodulatory effects.

## 2. A Brief History of Selenium Research

Though recent research has revealed the essential role of Se in immune system health, Se was not always recognized for its immunologically beneficial effects throughout history. In 1817, the Swedish chemist Jacob Berzelius first discovered Se and characterized many of its properties [8]. He named the element after the Greek moon goddess, Selene [9]. However, even during Berzelius’ experiments, he experienced hazardous health effects, including painful inflammation and breathing difficulties resulting from hydrogen selenide inhalation [8]. Since then, many other toxic effects of Se have been reported; thus, Se toxicity became the chief concern historically [10]. Adverse effects of “selenium poisoning” were documented in regions of the United States with high soil concentrations of Se [11]. One example is a 1943 report from South Dakota documenting adverse developmental effects experienced by growing chicks fed grains containing a novel toxicant, which were later reasoned to be attributed to high levels of Se [12]. Additionally, in Wyoming, there were anecdotal observations of selenosis reportedly causing substantial livestock deaths in the early 1900s [13]. Though no veterinary diagnoses were recorded at the time, there were later other veterinary-confirmed cases of selenosis occurring in Wyoming in the 1900s due to acute or chronic Se poising in livestock [13].

Interestingly, Se’s toxic properties were initially utilized to treat cancers, even in the early 1900s [14,15]. Se injections were also used in treatment protocols for different malignant diseases and pain management during that time [16]. However, it was not until further research was performed in the mid-1900s that the view of Se shifted from being primarily a toxic agent to having health benefits [10]. In 1954, Jane Pinsent published an important paper demonstrating the requirement of Se to produce a formic dehydrogenase enzyme in *Escherichia coli* [17]. Another landmark paper by Schwarz and Foltz in 1957 revealed the ability of Se to protect rats from lethal necrotic liver degeneration [18]. As a result of their work, Schwarz and Foltz deemed Se to be an essential trace element. These researchers also hypothesized that Se may play a role in reduction and oxidation (redox) reactions [18], a concept that would be confirmed and further developed in later years. In fact, it was not until 1973 that the Se’s redox mechanism of action was uncovered by Flohe, Gunzler, and Schock [19]. In this landmark paper, glutathione peroxidase (GPx), an enzyme known to combat lipid peroxidation, was first categorized as a selenoenzyme, meaning that Se is essential for normal GPx functioning [19]. Evidence of Se’s role in immune system regulation was revealed in the 2000s. One of these discoveries was the presence of selenoprotein K (SelK) in cells of the immune system (leukocytes) and its function as an endoplasmic reticulum membrane protein required for cell activation [20].

## 3. Environmental Biodistribution and Nutrikinetics of Selenium

### 3.1. From the Soil to the Plant

The first step of Se’s journey to the immune system begins in the soil. After elemental Se (Se^0^) is released into the ground, largely from the weathering of rocks, it is usually oxidized into inorganic selenite (SeO_3_^2−^, Se^+4^) or selenate (SeO_4_^2−^, Se^+6^). Organic forms of Se, namely selenoamino acids, can also be found in certain soils [1]. The chemical state in which Se is found is based chiefly on soil microbial activity, pH, redox properties, and other conditions [5]. In inorganic or organic states, Se can be taken up by plants into the chloroplasts via sulfur transport channels, phosphate transporters, and aquaporins [1,21,22]. Next, after a series of enzymatic reactions, Se is typically converted to selenide (Se^−2^) and reacts with serine to create selenocysteine (SeCys) [1]. Further enzymatic steps subsequently convert selenocysteine into selenomethionine (SeMet) [1]. In general, the Se in plants is generally found as SeMet, with lower levels of SeCys, selenate, and selenite present [22,23]. However, volatile forms of selenate and selenite can escape, especially after cereal grains have been dried and stored for multiple years, lowering the Se content of the plant by 4–73% [24]. This loss of volatile Se compounds is an important factor when considering the nutritive value of seleniferous crops. Another important distinction between Se-containing crops is their efficiency in acquiring Se. The plant families that accumulate organic Se to the greatest extent include the *Brassicaceae*, *Fabaceae*, and *Asteraceae* [21]. To help prevent human nutritional Se deficiencies, the strategy of fertilizing soils with inorganic Se is widely used around the world. This approach has proven successful for increasing Se concentrations in crops such as alfalfa, barley, perennial ryegrass, red clover, and lentils [1]. However, it does not come without risk, and Se soil concentrations should be monitored to avoid potential toxicity.

### 3.2. From the Water and Sediments to Plants and Algae

Just as terrestrial plants take up Se from the soil, aquatic plants and algae also acquire Se from water sediments and matrices. Rice, for example, which is typically planted and grown in flooded paddy fields [24], uptakes selenite, the predominant form of Se acquired from the environment, and converts it into SeMet just as terrestrial plants do. Algae are photosynthetic eukaryotic organisms that are also well known for their use of Se for growth and metabolic activities [25]. Due to the desire to enrich plant products, such as rice, with Se to overcome human dietary deficiencies, scientists have proposed several Se biofortification strategies. One such tactic involves using clustered regularly interspaced palindromic repeats (CRISPR)/Cas9 gene editing to increase Se accumulation, enhance Se transport, reduce Se volatilization, and improve Se metabolism in plants [24]. However, algae and other photosynthetic aquatic plants, including *Myriophyllum spicatum*, *Ceratophyllum demersum*, and *Potamogeton perfoliatus*, may suffer a decline in their photosynthetic abilities when exposed to high concentrations of Se [25,26]. Due to industrial pollution, Se toxicity in aquatic environments is a major concern, especially since it can bioaccumulate and biomagnify within aquatic ecosystems [27].

### 3.3. From the Plant and Algae to the Body

**Figure 2:**
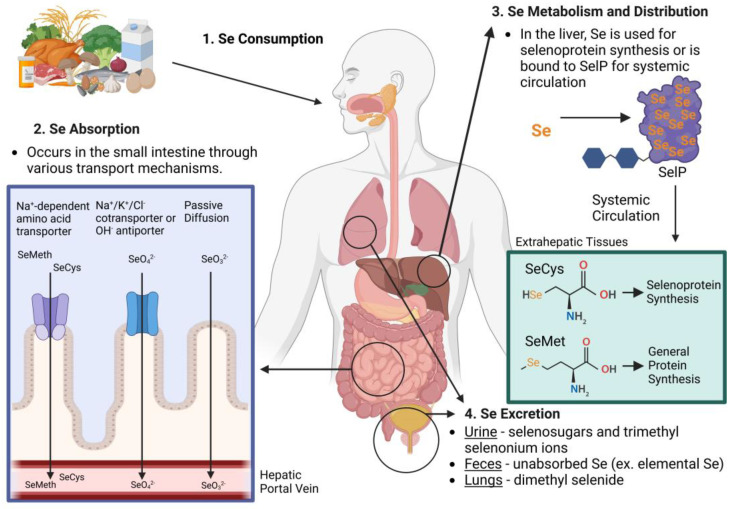
Absorption, distribution, and metabolism of selenium. Se: selenium; SeMeth: selenomethionine; SeCys: selenocysteine; SelP: selenoprotein P [21,28,29].

Se typically enters the human body through the ingestion of seleniferous plants. However, Se can also be consumed via algae, supplements, or certain animal products, as depicted in Figure 2. Examples of foods that are especially rich in Se include Brazil nuts, dairy products, meat, fish, cereals, breads, nuts, mushrooms, broccoli, garlic, onion, and other vegetables [21]. In these dietary sources, Se is found in a variety of forms, including SeMet, SeCys, selenate, and selenite.

Se is absorbed primarily through the small intestine via several mechanisms [28,30]. For organic Se, its absorption is carried out by a specialized Na^+^-dependent amino acid transporter [28]. The Na^+^/K^+^/Cl^−^ cotransporter or OH^-^ antiporter transports selenate, and selenite enters through Na^+^-independent passive transport [28]. Organic Se and selenate can both be relatively easily absorbed at a rate of 70–95%, but some studies have reported that selenite has a much lower absorption rate [28,29]. Sources of Se that are infrequently absorbed and utilized are Se dioxide, Se sulfide, and elemental Se [28]. Reportedly, Se nanoparticles have the highest bioavailability due to their miniscule size [31].

Se can be absorbed into the bloodstream as selenoamino acids, inorganic ions, and other forms [29]. Firstly, Se is transported to the liver where it can be utilized or bound within selenoprotein P (SelP) as SeCys residues for systemic circulation [29,30]. In circulation, SeCys or SeMet can be utilized by all tissues, either for incorporation into selenoproteins or general protein synthesis, respectively [6]. Notably, while SeMet can replace the typical amino acid methionine during protein synthesis, SeCys does not replace cysteine [23]. Instead, SeCys has been classified as the 21st amino acid as it is incorporated into elongating peptides once serine is enzymatically converted into SeCys on a transfer ribonucleic acid [29,32]. SeCys, but not SeMet, is used for the synthesis of bioactive selenoproteins [29]. The reason for the incorporation of Se in selenoproteins is due to the ability of Se to function in reversible reactions [33]. Moreover, enzymes utilizing SeCys instead of Cys can better withstand inactivation by oxidation, giving selenoenzymes a distinct advantage [33].

When not being used in selenoproteins, one important place for Se storage is the skeletal muscle, which reportedly stores approximately 28–46% of total Se in the body [9]. Other significant organs where Se accumulates to varying degrees in humans are the kidneys, liver, spleen, pancreas, heart, brain, lungs, and bones [9]. Se is even incorporated into hair at a rate of approximately 2.5 mg/kg, which is a much higher rate compared to the 0.1–0.8 mg/kg concentration found commonly across all internal tissues [6]. Though the incorporation of SeMet into proteins does not have any known biological relevance, many of these Se storage reservoirs are thought to be accessed during times of nutritional Se deficiency [28].

### 3.4. From the Body to the Environment

The main routes for Se elimination are via the feces and urine, with a small amount being released through sweat and the respiratory system [6,34]. In the urine, Se is typically lost from the body in the forms of selenosugars and trimethylselenoium ions [28]. However, the excretion of other Se metabolites, such as selenate, Se-methylselenoneine, methylselenocysteine, and L-selenomethionine, can also occur following the ingestion of a Se-rich meal or Se supplementation [6]. Volatile dimethyl selenide is the compound excreted via the respiratory system, and it has a distinctive unpleasant odor [28]; this process is enhanced when excessive amounts of Se are consumed [28]. Interestingly, organic forms of Se appear to be better retained by the body with lower excretory losses compared to inorganic Se [35].

## 4. Selenium’s Relevance to Human Health

### 4.1. Selenium Requirements

The Se requirements for males and females at different life stages can be seen in Table 1 [23]. First, for 0- to 6-month-old infants, their Se requirements are loosely established by estimating their daily consumption of Se in breast milk. Based on the results of various studies, the average concentration of Se in breast milk is said to be approximately 18 µg/L in Canada and the United States. Though there is some consistency in the milk Se concentration in non-supplemented healthy mothers, milk Se levels can still fluctuate depending on the mother’s dietary Se consumption. Next, the Se recommendation of 20 µg/day for infants aged 7 to 12 months was extrapolated from the 0- to 6-month-old’s Se requirement of 15 µg/day and verified using an estimation of consumed Se in breast milk and weaning foods. For children from 1 to 3, 4 to 8, and 9 to 13 years of age, requirements of 20, 30, and 40 µg/day, respectively, were extrapolated from adult values [23].

In order to establish a dietary reference for adult Se intake, intervention studies from China and New Zealand were analyzed [23]. The 1987 Chinese study concluded that a daily Se intake of 41 µg SeMet was sufficient to maximize plasma GPx activity, and higher supplementation rates had no significant benefit [23,36]. By multiplying by a weight adjustment factor to compensate for the heavier weights of North American males in comparison to Chinese males, a dose of 52 µg Se/day was recommended for North Americans [23]. However, the findings of the 1999 New Zealand study slightly modified this recommendation [37]. Analyses of this study’s data by an independent group suggested that only 38 µg of Se per day was needed due to a lack of significant differences between the increases in GPx activity among the supplemented groups [23]. This led to the estimated average Se requirement to be calculated as 45 µg ([38 µg from the New Zealand study + 52 µg from the Chinese study] ÷ 2) [23,36]. To calculate the recommended dietary allowance, a coefficient of variation of 10% was added twice to the estimated average requirement to take into consideration the needs of a wider range of individuals in the adult age range (19–50 years). This yielded a Se requirement of 55 µg (45 µg + [5 µg × 2]) for both adult men and women [23]. One exception is the Se recommendations for pregnant and lactating women, which are slightly higher due to the presumed need for maternal Se transfer to the fetus and neonate [23]. The Se requirements do not change for older adults due to the lack of evidence demonstrating that aging alters either Se metabolism or utilization [23].

Both the New Zealand and Chinese studies that were used to establish these adult Se requirements, however, have several limitations. Firstly, for the Chinese study, only graphed results were used to interpolate the conclusions as the primary data were unavailable. This raises concern about data and study reliability and transparency. Moreover, no women were included in the study. Therefore, any effects that biological sex differences may have on Se metabolism or utilization were not accounted for. Additionally, since only people of Chinese descent participated in the study, this raises the question of whether these results are transferable to all populations. There were also only eight or nine men per Se supplementation group, which is a small sample size to draw from considering that these results were used for establishing Se requirements worldwide. Moreover, GPx was the only selenoprotein assessed for optimal activity in this study. However, other selenoproteins, such as SelP, may need higher concentrations of dietary Se to be optimally expressed [38,39]. Further research should be conducted to verify these results by assessing the activation of additional selenoproteins and using a larger and more diverse sample population [37]. Secondly, the New Zealand study also had a few limitations [37]. As in the Chinese study, the basal dietary Se concentrations were only estimated and not verified, revealing a lack of control over how much Se the participants consumed in their daily diets. Consequently, this could be a confounding factor adding uncertainty to their conclusion. Next, in their study design, their highest supplementation dose was 40 µg of SeMet per day in addition to the estimated 30 µg of Se per day from the basal dietary levels. Therefore, these researchers did not test any supplemental levels that were above the highest recommended dietary allowances at the time (70 µg of SeMet per day). The effect of higher rates of supplementation should have been investigated to determine whether they improved GPx activity significantly. Additionally, only SeMet was studied, and not any other sources of inorganic or organic Se, which may have significantly affected the recommendation. Moreover, there was no citation provided for the independent analysis of the study data, which appeared to draw considerably different conclusions from the original study [23]. As previously mentioned, independent analyses of the data led to the conclusion that 38 µg of Se per day was sufficient for intake. Contrarily, the researchers in the original article found that the participants consuming approximately 70 µg of SeMet per day reached a plateau in their GPx activity, while the other supplemented groups did not. Consequently, they concluded that 70 µg of Se per day was the optimal upper-level Se requirement needed to maintain health, with lower levels being suitable to partially activate plasma GPx functions [37]. Thus, more research should be conducted to confirm the results of this study and to explain why there was a difference found by the group who independently analyzed the data.

### 4.2. Se Deficiency and Risk of Disease

If Se requirements are not met through diet or supplementation, Se deficiency may become a problem. There are many adverse health events linked to inadequate Se intake including, but not limited to, increases in cancer incidence and pathogenic viral mutations due to excess reactive oxygen species (ROS) production, deteriorations in cognitive function, an increased risk for coronary heart disease, reduced fertility, attenuated immune responses, and impaired thyroid function [40]. Kashin–Beck disease is also suggested to be caused partly by a Se deficiency; people suffering from this disease develop chronic and disabling osteoarthropathy [40]. The most notable condition indicating Se deficiency is Keshan disease, a cardiomyopathy causing the enlargement of the heart that may also be linked to Coxsackie B viral infection [23,40]. Non-supplemented people who live in Se-deficient geographical regions where Keshan disease is endemic have much lower plasma Se, GPx, and SelP concentrations compared to supplemented individuals in the same region [23]. To prevent the development of this disease, multiple studies from China, New Zealand, and Finland have demonstrated that an intake of Se above approximately 20 µg per day is adequate [23]. However, simply consuming 20 µg of Se per day will not optimize the activity of all selenoproteins throughout the body and may not prevent other Se-related deficiency conditions.

People who live in geographical regions with low soil Se concentrations are at a higher risk of developing Se deficiency or a suboptimal Se status. Several regions in China and Tibet are especially well known for their Se-depleted soils, and without supplementation, people from these regions will experience Se deficiency [28]. One systematic review analyzing data from countries in the Middle East and Europe also found that some people living in places such as Eastern Europe, Slovenia, Turkey, Saudi Arabia, and Italy had lower Se statuses than what is required to optimize GPx activity in the blood [41]. However, there is some disagreement in the literature regarding what plasma Se concentrations are needed to optimize the activity of plasma GPx. The values recommended for ideal plasma Se concentrations range from 63.16 µg/L to 98.7 µg/L [41]. Overall, more research needs to be conducted to establish what constitutes an optimal Se status so that Se deficiency can be avoided.

### 4.3. Excess Consumption of Se and Risk of Toxicity

On the other hand, the excessive intake of either organic or inorganic Se can be just as harmful to human health, though it is far less prevalent in human populations compared to Se deficiency [28]. The main routes of exposure by which Se toxicity can occur are via inhalation or ingestion. One notable symptom of Se toxicity is a distinctive garlic odor from the breath, which is due to the lungs expiring the volatile Se metabolite dimethylselenide [42]. An association between a high Se intake and the risk of diabetes mellitus has also been reported in a meta-analysis [43]. Though the precise cause of diabetes remains unknown, some researchers propose that dietary Se excess may increase the circulation of reactive selenocompounds that impair proper signaling and metabolic processes [44]. Other common symptoms of Se toxicity include gastrointestinal disturbance, respiratory distress, hair loss, nail deformation, and neurological issues; however, more research is needed to precisely define the mechanisms through which Se induces these symptoms [45].

Oil refinery effluent, agriculture drainage wastewater, and electric power plant aqueous runoff can all contain high levels of Se [26], and people experience Se poisoning when ingesting water or food sources from environments with high Se levels. For example, in the 1960s, people living near coal deposits in certain rural communities of the Chinese Hubei and Shanxi provinces experienced many of these symptoms due to their consumption of crops grown on extremely Se-rich soils [42]. Their blood Se concentrations ranged from 1.3 to 7.5 mg/L, and their daily dietary Se intakes ranged from 3.2 mg to 6.8 mg, which is much higher than the recommended value [42].

Because of the negative side effects experienced by Chinese patients with selenosis, the Institute of Medicine established an upper limit of 400 µg per day for Se intake in adults, as seen in Table 1 [23]. However, one study found an all-cause mortality hazard ratio of 1.62 in a Se-supplemented group receiving 300 μg of Se per day over 5 years compared to the placebo group [45]. On the other hand, the daily consumption of 724 µg of Se has also been reported without any adverse health effects, suggesting that the amount of ingested Se needed to elicit toxic effects may be much higher in some individuals [23]. One explanation for these variations could be genetics. Some studies indicate that the presence of single-nucleotide polymorphisms (SNPs) in genes related to Se metabolism, utilization, and excretion can play a role in how much Se a person can tolerate before experiencing toxic effects [46,47]. For example, Se can be excreted as trimethyl-selenonium ions in the urine of people who have AA or AG *indolethylamine N-methyltransferase* SNP genotypes, possibly helping them remove excess Se more efficiently than those with other genotypes [46]. Nevertheless, there is still uncertainty regarding what the daily maximum of Se intake should be to avoid selenosis and whether this upper limit should be adjusted depending on the population.

On the contrary, there are times when ingesting high levels of Se may be beneficial to human health. As recently reviewed, high levels of Se consumption may be advantageous to combat the effects of arsenic, cadmium, or mercury poisoning [48,49]. Some mechanisms of action through which Se is proposed to act are via binding to these heavy metals to create biologically inert compounds, increasing the methylation and subsequent excretion of these toxic metals, and enhancing antioxidant activity to protect against biological damage elicited by heavy metals [48]. Since heavy metal toxicity is a concern in our industrialized world, more research should be undertaken to assess how different Se compounds may be therapeutically applied to mitigate the toxicity of heavy metals.

One form of Se that is substantially less toxic than inorganic Se sources is Se nanoparticles, which can be biosynthesized in a safe, ecologically friendly manner by bacteria, fungi, plants, and algae [31]. Interestingly, their ROS-inducing, apoptosis-promoting, and immunostimulatory, targeted toxic effects are carried out primarily against cancerous cells, and not normal healthy cells, making Se nanoparticles a biologically safe and more effective alternative to organic and inorganic Se. One proposal as to why Se nanoparticles selectively induce these deleterious mechanisms of action in cancer cells and not healthy cells is due to the imbalanced redox levels and acidic pH found in cancer cells [31].

## 5. The Impact of Se on the Immune System

### 5.1. Antioxidant Activity

**Figure 3:**
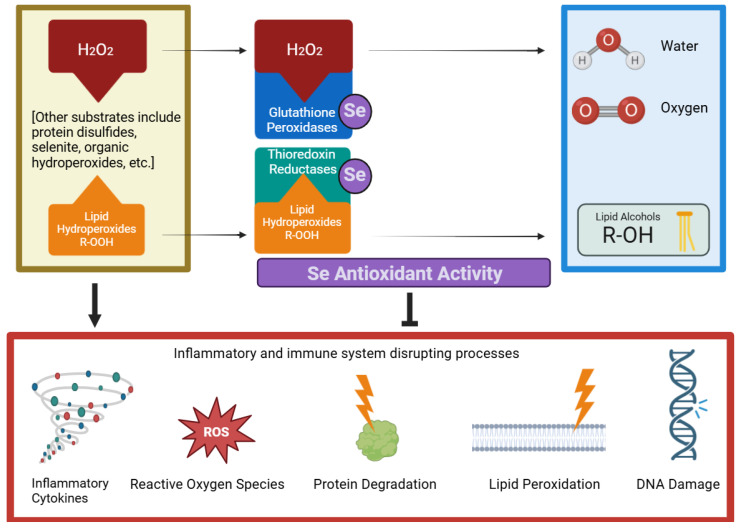
Antioxidant activity of selected selenoproteins [52].

Antioxidant activity is one of Se’s most widely known beneficial properties, as depicted in Figure 3. This antioxidant activity protects cells from oxidative damage and promotes their normal function. While these antioxidative processes happen throughout the body, analyzing how antioxidant defense mechanisms specifically affect the immune system will be the focus of this section. Maintaining an appropriate level of ROS and reactive nitrogen species (RNS) is essential to prevent oxidative damage in healthy tissues and promote optimal immune responses [50]. While the presence of some ROS is required for initiating T cell activation and differentiation, apoptosis, the elimination of pathogens, and other cellular signaling activities, antioxidants are needed to deal with the excessive production of these potentially damaging free radicals [50]. Excessive free radicals can cause lipid peroxidation of host–cell membranes, DNA damage, and protein degradation, which can profoundly affect numerous physiological processes, including immune function [51]. Without proper antioxidant regulation, valuable leukocytes and lymphoid tissues can be damaged; thus, antioxidants like Se are required to help regulate the immune system’s activities.

Many selenoproteins function in an antioxidant capacity throughout the body, as demonstrated in Figure 3 [52]. These include GPxs 1-–4 and 6 and thioredoxin reductases (TrxRs)1–3 [50]. GPx5 and GPx7–8 do not contain selenocysteine residues; they only have a cysteine amino acid in the active site and do not require Se for functioning [50]. Broadly speaking, these antioxidant selenoproteins inactivate damaging free radicals through reduction reactions. For example, GPxs catalyze the conversion of reactive hydrogen peroxide (H_2_O_2_) into water and oxygen [52]. Additionally, GPx4 reduces membrane lipid hydroperoxides, and TrxRs reduces protein disulfides [52]. However, when such antioxidant processes are insufficient or overwhelmed, an individual’s risk for different pathological conditions and diseases increases [52]. These include cancers, neurodegenerative diseases, fertility issues, renal impairment, cardiovascular diseases, muscle disorders, and many others [52].

One immunological benefit of Se’s antioxidant activity is its attenuation of inflammatory processes caused by ROS/RNS. The improper regulation of leukocytes and disease progression can lead to excessive ROS/RNS during inflammation, which is damaging to tissues. Therefore, antioxidant control of these processes by selenoenzymes is pertinent. One study using a porcine model found increased antioxidant capacity in animals receiving organic Se in their diets [53]. One of the main findings of this study was that the organic Se supplement, 4-methylselenobutanoic acid (HMSeBA), increased the expression of GPxs and TrxRs in the duodenum, jejunum, ileum, colon, and thymus. The level of malondialdehyde (MDA), a biomarker of lipid peroxidation, also demonstrated a decreasing trend in most of the gastrointestinal tissues of the organic Se-supplemented gilts compared to the control. Thus, animals provided with an HMSeBA-enriched diet may combat ROS/RNS-induced oxidative damage and inflammation more efficiently, promoting improved immune function compared to the non-supplemented control group. Systemically, an anti-inflammatory effect was reflected by the attenuated baseline levels of serum pro-inflammatory cytokines IL-6 and tumor necrosis factor α (TNF-α) and elevated baseline levels of the anti-inflammatory cytokine IL-2 in the organic Se-supplemented gilts compared with the control animals. Overall, this antioxidant activity improved by Se lowered baseline inflammatory cytokine activity and reduced lipid peroxidation, which are both vital to prevent oxidative damage, inflammatory pathologies, and tumorigenesis [53]. Hence, organic Se supplementation may confer immunologically beneficial antioxidant effects on gilts. However, more research involving a pathogen challenge should be conducted to verify how Se specifically modifies the immune response.

Studies with chickens also indicate that Se may have beneficial immunomodulatory and antioxidant activities. For example, Se-deficient broiler chickens had a significant decrease in 22, 20, and 23 selenoproteins expressed in the thymus, spleen, and Bursa of Fabricius, respectively, in comparison to chickens supplemented with 0.2 mg/kg of Se [54]. Many of these selenoproteins, such as the GPxs, TrxRs, and iodothyronine deiodinases (Idios), play an important role in the antioxidant defense activity of these lymphoid organs [54]. Moreover, supplementation with Se yeast also caused a corresponding elevation in the expression of pertinent antioxidant selenoprotein genes in the intestines of laying hens compared to the Se-deficient control group [55]. These included *GPX1*, *GPX3*, *GPX7*, *GPX8*, *SELENOM*, *SELENOT*, and *SELENOW*, which all function to regulate redox balance [55]. In this way, Se may improve intestinal health, reduce unnecessary inflammation, and help to maintain the homeostasis of different cellular metabolic processes.

Supplementing fish with 1 mg of organic, inorganic, or elemental nano-Se per kg of diet during a feeding trial also showed beneficial antioxidant effects [56]. For example, the serum MDA activity decreased equally across all Se supplementation groups compared to the Se-deficient control group, revealing the improved antioxidant status of the fish provided with Se. Additionally, the basal serum concentrations of GPx and the non-Se-dependent antioxidant enzymes catalase (CAT) and superoxide dismutase (SOD) were significantly higher across the treatment groups in comparison to the Se-deficient control group. Most notably, the groups supplemented with elemental Se nanoparticles had the highest concentrations of serum antioxidant enzymes. Overall, this study highlighted the potential benefits of Se supplementation, especially nano-Se, in reducing oxidative stress in fish.

However, other researchers found different, dose-dependent consequences pertaining to oxidative stress after providing European Sea Bass with dietary Se nanoparticles at rates of 0, 1, 5, or 10 mg/kg [57]. In this study, the activities of SOD, CAT, and GPx in the serum were all significantly enhanced in the fish that received 1 mg of Se/kg. Contrarily, at dietary Se inclusion rates of 5 mg/kg and 10 mg/kg, the activities of these enzymes either significantly decreased or did not change relative to the control group. Furthermore, the serum MDA concentrations significantly decreased when Se was provided at concentrations of 1 mg/kg or 5 mg/kg of diet compared to the control, while the MDA levels increased substantially when 10 mg/kg was added to the diet. Therefore, while 1 mg of Se/kg appeared to have protective effects against lipid peroxidation and oxidative stress, administering 10 mg of Se/kg had detrimental effects on fish antioxidant activities, showing the need to carefully evaluate the dosing regimen of different species when considering Se supplementation.

In conclusion, though the antioxidant functions of Se may be able to beneficially modulate the immune system through scavenging ROS and attenuating inflammatory processes, more high-quality research in humans is needed before effectively implementing Se at the clinical level [58,59]. Its potential to regulate cytokine production to maintain homeostasis also remains an important feature that should continue to be investigated [59].

### 5.2. Innate Immune Response

One defining characteristic of innate immunity is its rapid response to infection. To control and eliminate pathogens, the innate immune system uses complement system activation, activated phagocytic cells, and antimicrobial peptides [60]. Cells involved in the innate response include mast cells, natural killer (NK) cells, monocytes, macrophages, dendritic cells, neutrophils, basophils, and eosinophils. A primary means by which these leukocytes kill pathogens is through the generation of ROS during a biochemical reaction referred to as “oxidative burst”, a process regulated in part by selenoproteins such as SelK and GPx that assist in the Ca^2+^ and redox signaling pathways, respectively [61]. While the antioxidant mechanisms of Se control the excessive production of ROS that can damage host tissues, adequate Se intake also improves the efficacy of the oxidative burst initiated in phagocytes stimulated by a pathogen. In Se-deficient conditions, this oxidative burst process is impaired, but at increasing Se levels, this oxidative burst simultaneously heightens, even though Se’s antioxidant activities elsewhere throughout the body concomitantly increase [61]. These ROS-stimulating and attenuating processes of Se are independent, as one does not interfere with the functioning of the other. This enables the immune system to effectively destroy microbes when encountered and ensure that the ROS generated do not cause excessive host tissue damage. Additionally, leukocytes depend on Se regulation for survival and differentiation. For example, Se promotes the differentiation of pro-inflammatory M1 macrophages to anti-inflammatory M2 macrophages [62]. Moreover, Se enables the Ca^2+^ signaling that is required to initiate the macrophage FcγR receptor-mediated phagocytosis of IgG-opsonized microbes [61]. Se supplementation may even reduce leukocyte adhesion to endothelial cells, which would presumably influence their translocation into various tissues [61]. Overall, Se has many mechanisms of modulating innate immune function.

Key studies evaluating the interactions between Se and the innate immune system will now be highlighted to gain a deeper understanding of Se’s immunomodulatory role. One previously mentioned role of Se is its incorporation into SelK, which is embedded in the endoplasmic reticulum of leukocytes like neutrophils and macrophages. SelK contributes to the influx of Ca^2+^ needed for cellular signaling during immune responses [20]. Researchers found evidence of this when comparing wild typewild type versus *SELENOK* knockout mice. Regarding the innate immune system function, *SELENOK* knockout mice displayed attenuated neutrophil migration and production of chemokines. *SELENOK* knockout mice also had impaired macrophage Fcγ receptor-mediated oxidative bursts and an attenuated secretion of Toll-like receptor (TLR)-induced cytokines IL-6 and TNF-α in comparison to the wild type mice [20].

In another study, researchers investigated the effects of Se and probiotics on Alzheimer’s disease [63]. Though the purpose of this study was not to test the impact of Se supplementation on the innate immune system, the results coincidently revealed a couple of Se’s immunomodulating properties. In this randomized double-blind controlled trial, the patients consumed 200 µg of Se per day for 12 weeks. Compared to the non-supplemented control group, Se supplementation significantly decreased the concentration of circulating C-reactive protein (CRP), which is an acute-phase inflammatory protein that contributes to the innate immune response in part by activating the classical complement pathway after ligating to C1q [64]. This outcome is especially beneficial in terms of treating inflammatory conditions like Alzheimer’s disease or rheumatoid arthritis. Additionally, the plasma levels of low-density lipoprotein (LDL) cholesterol were significantly lower in the Se-supplemented group compared to the control group. Hypercholesterolemia causes cholesterol accumulation in macrophages, enhances TLR signaling, elevates blood monocyte and neutrophil numbers, and increases inflammatory cytokine production. Therefore, Se’s ability to lower blood LDL cholesterol levels also contributed to attenuating innate inflammatory responses. This regulatory action may be immunologically advantageous for those with autoimmune diseases, inflammatory disorders, or chronic infections [65]. Overall, Se demonstrates promising capabilities in modulating the innate immune system by decreasing the blood concentrations of CRP and LDL cholesterol.

In a fish study, Se-deficient Nile Tilapia were provided 1 mg/kg of inorganic Se, equivalent levels of organic Se, or elemental Se nanoparticles [56]. In these Se treatment groups, the biomarkers implicated in innate immune function were higher in comparison to those in the Se-deficient control group. For example, the activity of serum lysozyme, a key antimicrobial enzyme, was significantly higher in the nano-Se and inorganic Se supplementation group compared to the other treatment groups. Moreover, the nano-Se-supplemented fish performed significantly better in their nitro blue tetrazolium tests due to their phagocytic oxidative burst strength and bactericidal killing of *Aeromonas hydrophila*, which are both indicators of enhanced innate immunity. There was also a significant increase in the baseline pro-inflammatory cytokine mRNA expression relevant to the innate immune system, including *TNFα*, transforming growth factor beta 1 (*TGFβ1*), and interleukin-1 beta (*IL1β*) in the nano-Se supplemented group in comparison to the control group, thus revealing Se’s immunostimulatory capabilities. However, there were no changes in the circulating concentrations of leukocytes such as monocytes, eosinophils, and basophils. Overall, nano-Se supplementation had the most pronounced benefit on the innate immune system of Se-deficient fish. Therefore, different supplementation rates of nano-Se could also be investigated for use in people to improve their innate immunity.

In another fish study with similar findings, European Sea Bass were administered Se nanoparticles at dietary inclusion rates of 0, 1, 5, or 10 mg/kg [57]. Interestingly, whether Se promoted or attenuated the muscle tissue’s basal expression of cytokines critical to innate immunity partially depended on the concentration of Se included in the diet. For example, the relative expression of *IL6* decreased significantly when the fish were given 1 mg of Se/kg in their diets, but expression was significantly enhanced when the fish were administered 5 or 10 mg of Se/kg in their diets. IL-6 is an important modulator of the innate immune response in both fish and humans as it triggers necessary immunomodulatory processes, such as the acute phase response, complement activity, antimicrobial peptide production, and phagocytosis [57,66]. However, chronically elevated levels of IL-6 can contribute to several types of autoimmune disorders, such as rheumatoid arthritis, autoimmune epilepsy, and Neuromyelitis Optica spectrum disorder. Therefore, whether or not humans have the same dose-dependent responses to differing Se concentrations regarding their *IL6* expression would be a worthy investigation, as attenuating the basal production of certain cytokines may be therapeutically valuable. Additionally, the gene expression of the pro-inflammatory *TNFα* cytokine, which is involved in many facets of the immune system, increased across Se treatment groups in comparison to the control. Finally, basal *IL12* expression was significantly enhanced in the muscle tissue of the fish at the 1 mg of Se/kg dietary inclusion rate but remained the same at the 5 mg/kg and 10 mg/kg inclusion rates. IL-12 is a cytokine that helps to facilitate communication between the innate and adaptive immune systems [67]. It is secreted by professional antigen-presenting cells, including dendritic cells and B cells, and phagocytes like macrophages, granulocytes, and monocytes in response to TLR–pathogen interactions [67]. The main targets of IL-12 are T cells and NK cells, as this signaling enhances interferon gamma (IFN-γ) and subsequent T helper 1 cell differentiation [67]. High levels of IL-12 can be associated with inflammatory conditions such as psoriasis and multiple sclerosis, thus showing the need to regulate its production [67]. Overall, since the different levels of nanoparticle Se supplementation had different effects on immune system parameters, the immunosuppressing or immunostimulatory consequences of Se administration may largely be dose dependent. From these studies, Se evidently plays a role in modulating the immune system, and this property may be harnessed in human dietary interventions to improve health or disease recovery once further research is conducted to optimize dosing regimens.

### 5.3. Adaptive Immune Response

Another prominent feature of Se is its ability to modulate the adaptive immune system. Broadly speaking, the cellular branch of the adaptive immune system uses T cells, while the humoral branch uses B cells. The immunomodulatory mechanisms of Se have been previously reviewed elsewhere [61]. Generally, in addition to Se’s ability to regulate ROS signaling for oxidative burst and increase protein biosynthesis, Se also elevates the production of the cytokines IL-2 and IFN-γ by T cells, which promote T cell proliferation and differentiation, influence epigenetic regulation, prevent endoplasmic reticulum stress, and reduce leukocyte tissue infiltration. In this section, relevant studies will be reviewed to update what is known about Se’s impact on the adaptive immune system. 

One selenoprotein that plays a part in the adaptive immune system is SelK. SelK can be found in the endoplasmic reticulum of B and T cells. As previously highlighted, SelK supports the Ca^2+^ flux needed for cellular signaling and activation [20]. Thus, in the study by Norton et al., it was not surprising to observe that CD4^+^ and CD8^+^ T cells in *SELENOK* knockout mice had significantly reduced proliferation and migration compared to the wild type mice. This study demonstrated the essentiality of Se for the proper function of the cellular adaptive immune response.

Another Se study using a murine model looked at how placental cytokine concentrations altered after maternal immune activation [68]. In this study, the mice consumed Se from gestation day 9 until birth and were challenged with polyinosinic:polycytidylic acid on gestation day 17 [68]. In comparison to the mice who did not receive Se, the mice who received Se daily had significantly attenuated IL-17 and IL-1β protein concentrations in their placental tissues; however, the IL-6 levels remained unchanged. IL-17 and IL-1β are important pro-inflammatory mediators primarily secreted by Th17 cells and monocytes or macrophages, respectively [69]. Therefore, this study showed Se’s ability to attenuate the activities of the cell-mediated branch of the adaptive immune system as well as parts of the innate system within the context of pregnancy, indicating that Se’s anti-inflammatory and immunomodulatory effects may even be beneficial to the fetus during critical developmental periods.

An additional study using a porcine model found some interesting results related to Se’s ability to improve the humoral branch of the adaptive immune system [53]. These researchers assigned gilts to different dietary treatment groups: Se-deficient control, 0.3 mg/kg of feed sodium selenite, and 0.3 mg/kg of feed HMSeBA. They found that the intestinal immunoglobulin A (sIgA) and serum IgG concentrations were significantly elevated in the HMSeBA-treated gilts in comparison to the control animals. The production of antibodies is an important indicator of the productivity of differentiated B cells; therefore, this study illustrated the requirement of Se for normal humoral adaptive immune responses.

Other researchers used chickens to study the effects of Se on the adaptive immune system. In groups of chickens consuming different forms of Se (sodium selenite or various Se-enriched yeast strains) at a rate of 0.3 mg of Se/kg of basal diet, there were distinct effects noted in terms of the adaptive immune system [70]. Notably, the total number of white blood cells was significantly lower in most of the Se yeast supplementation groups compared to the control group receiving the basal diet; however, by day 42, the serum concentrations of IgG, IgA, and IgM increased significantly across all Se supplementation groups [70]. Another research group also compared the immunological impacts of dietary organic Se yeast to inorganic sodium selenite using chickens [71]. These researchers vaccinated chickens with an inactivated low-pathogenicity avian influenza virus to assess antibody production using birds that were fed organic or inorganic Se at a rate of 0.15 mg/kg or 0.30 mg/kg of basal diet. Though organic Se supplementation increased the production of antigen-specific antibodies in comparison to the inorganic Se supplementation group, there was no significant difference in the antibody titres between the Se-supplemented and the non-supplemented control birds. There were also no significant differences in the production of IgM and IgY across the treatment groups. However, both 0.15 mg/kg and 0.30 mg/kg of organic Se supplementation displayed significantly attenuated viral shedding from the cloaca and oropharynx after a challenge with the same pathogen. Therefore, though Se supplementation did not have a notable effect on antibody production, Se supplementation exhibited the ability to reduce viral shedding, possibly revealing its antiviral properties [71]. Another broiler chicken study supplemented the birds with 0, 0.25, 0.50, or 1.00 mg of Se/kg and challenged them with *Clostridium perfringens* to induce necrotic enteritis. Interestingly, Se had an immunostimulatory effect on these birds by significantly enhancing the expression of jejunal and splenic *IL1β*, *IL6*, and *IL8* [72]. Though the body weight gain and the blood concentrations of necrotic enteritis B-like toxin antibodies also increased significantly in some Se-supplemented groups, there were no significant differences in the intestinal lesion scores. Overall, Se supplementation proved to be moderately beneficial for promoting the humoral immune response in chickens.

Finally, studies with fish also showed some notable improvements in the adaptive immune system due to Se supplementation. In a feeding trial, Nile Tilapia were administered 1 mg of inorganic Se/kg, 1 mg of organic Se/kg, or 1 mg of elemental nano-Se/kg [56]. The nano-Se treatment displayed a significantly higher total IgM, while the organic Se treatment displayed significantly higher serum protein levels compared to the control group that received the basal diet. Moreover, another study showed that fish supplemented with nano-Se at a rate of 0, 1, 5, or 10 mg/kg of basal diet had significantly increased levels of IL-2, a cytokine responsible for limiting inflammation by promoting T-regulatory cell development, across nano-Se treatments compared to the control group, but the response was not dose dependent [57,73]. Overall, all these animal studies have shown promising results regarding Se’s positive impact on the immune system. These findings should encourage further research in people to see how Se may be utilized to improve adaptive immunity.

### 5.4. Gut Microbiota

Human microbiota contains a multitude of microorganisms, such as bacteria, viruses, fungi, and parasites, that colonize the skin, gut, and other mucosal surfaces [74]. Evaluating how micronutrients like Se alter gut microbiota is a relatively new and exciting area of research. Selenoprotein genes can be found in approximately a quarter of all bacteria [75]. Some of these include *E. coli*, *Clostridia*, and *Enterobacteria* classes found within gut microbiota [75]. Therefore, either an increase or decrease in dietary Se intake will likely also modulate metabolic activities and the abundance of bacterial species. However, the concomitant use of Se by microbiota can sometimes detract from the host’s supply during limited Se intake, creating competition for Se between the host and microbiota. Supplementation with Se may even promote the colonization of pathogenic bacteria if they can utilize Se before it can be absorbed by the host [76]. Contrarily, the metabolism of Se by the host and host microbiota can also be symbiotic, especially as certain microorganisms biotransform inorganic Se to organic forms that are more readily absorbed by the host [75]. Therefore, the relationship between the host, microbiota, and Se is complex; however, most evidence points towards Se supplementation as a beneficial strategy to promote a healthy gut microbiome. Furthermore, it is now widely accepted within the scientific community that gut microbiota impact the host immune response. While this topic is reviewed in depth elsewhere [74], the goal of this section is to highlight the particular role of Se in modulating the composition of gut microbiota and its subsequent effect on the human immune system.

One important murine study investigated the effect of nutritional and supranutritional Se supplementation on the gut microbiota composition [77]. An advantageous feature of this study was that the researchers used nutritional Se concentrations that mimicked a person’s daily Se consumption to make the study more relevant to humans. In this study, the researchers found a significantly increased relative abundance of certain bacterial strains, including those of the *Akkermansia* and *Turibacter* genera, in supranutritional Se-supplemented mice that were fed 0.40 mg of sodium selenite/kg of feed compared to the Se-deficient control mice. These bacterial genera, especially *Akkermansia,* are known to play a role in beneficial processes such as immunomodulation, anti-inflammatory activity, and disease resistance [78,79]. Both the nutritional and supranutritional Se-supplemented mice also had a reduced abundance of *Dorea*, a bacterial strain whose increased abundance is linked to various diseases such as autism spectrum disorders and irritable bowel syndrome [80,81]. Additionally, significantly higher GPx activity in the liver and higher serum Se concentrations were noted in the nutritional and supranutritional Se-supplemented groups in comparison to the Se-deficient control group, indicating enhanced antioxidant potential for combatting oxidative stress. Overall, the results of the first portion of this study provide strong evidence of the microbiota-modulating activity of nutritional and supranutritional levels of Se, with a potentially greater benefit when supplementing at supranutritional Se levels. More research should be conducted to confirm this and further establish effective supplemental Se dosing guidelines to promote healthy gut microbiota.

In the next portion of Zhai et al.’s study, fecal microbiota transplantation (FMT) was performed using donor fecal matter from Se-supplemented and Se-deficient control mice, and the recipient mice were non-supplemented mice; this FMT was performed to assess whether the Se-modulated microbiota would impact the recipient mice’s immune responses and health. Interestingly, the expression of notable epithelial tight junction proteins Zonula occludens-1 and Claudin-1 were increased in the recipient mice that received FMT from Se-supplemented mice, indicating that they had improved gut barrier function in comparison to the recipient mice that received FMT from the Se-deficient mice. Promoting proper gut barrier integrity is essential for preventing gastrointestinal pathogens from circulating systemically and is therefore an important mechanism for disease prevention [82]. Additionally, there were increased levels of sIGA and reduced concentrations of inflammatory cytokine IL-1β in the colons of mice that received FMT from Se-supplemented mice, again highlighting Se’s beneficial effects on the gut microbiota and indirect benefits to the host’s immune function. Another primary point of comparison between the FMT treatment groups was the concentration of colonic short-chain fatty acids (SCFAs). Both Se-supplemented FMT recipient groups had significantly higher concentrations of SCFAs, such as acetic acid, propanoic acid, and butanoic acid, in their colons compared with the Se-deficient FMT recipient group. While a comprehensive review of how SCFAs influence the microbiota and immune system is outside the scope of this review, there is strong evidence that SCFAs are an energy source for intestinal epithelial cells and have beneficial immunoregulatory properties [83].

Finally, after the FMT recipient mice from the Zhai et al. study were challenged with dextran sulfate sodium (DSS), other biochemical and physical parameters were also altered. For example, after the FMT mice were treated with DSS, the supranutritional Se FMT recipient mice had significantly lower colitis damage scores and serum endotoxin concentrations in comparison to the Se-deficient FMT recipient mice, likely attributed to enhanced gut barrier function [84]. Additionally, the researchers noted significantly reduced colonic IL-1β, IL-6, IL-8, and TNF-α concentrations in either one or both Se-supplemented FMT recipient groups after the DSS challenge, indicating attenuated levels of harmful inflammation compared to the Se-deficient FMT recipient group. Additionally, following a *Salmonella typhimurium* pathogen challenge, colonic IL-1β, TNF-α, and IL-6 concentrations were significantly attenuated in one or both Se-supplemented FMT recipient groups in comparison to the Se-deficient FMT recipient group. Moreover, reduced numbers of bacterial colonies were found in the liver and spleen, and the serum endotoxin levels were significantly lower, indicating that the Se-modulated microbiota helped to mitigate the systemic spread of *S. typhimurium*. Overall, the results from both the DSS and *S. typhimurium* challenges well support the promising role of Se in beneficially modulating the gut microbiota to support the health of the host.

Another murine study also demonstrated the capacity of Se to modulate the gut microbiota and possibly some colonic cytokine activity [85]. The mice in this study were fed either Se-enriched (1 mg/kg of feed) or Se-depleted (0.1 mg/kg of feed) versions of a high-fat/high-sugar or a low-fat/low-sugar diet for 28 days. One significant finding of this study is that the expression of inflammatory cytokines *TNFα* and *IL16* in the caecum was significantly reduced in the high-fat/high-sugar mice compared to that in the mice receiving a low-fat/low-sugar Se-enriched diet, showing that Se may have had anti-inflammatory effects in the presence of a high-fat/high-sugar diet. However, there were no significant differences in the expression of inflammatory or anti-inflammatory cytokines in either the ileum or the caecum when comparing the Se-enriched diet to the Se-deficient diet. In the ileum, a statistically significant increase in *Peptostreptococcaceae* was noted with the Se-enriched high-fat/high-sugar diet in comparison to the Se-depleted high-fat/high-sugar diet. *Lactobacillaceae* also increased from the Se-depleted to the Se-enriched version of the high-fat/high-sugar diet, but the increase was not statistically significant. The authors did not perform any immunological challenges on the mice, so the true relevancy of these slight microbiome changes to murine health remains unknown. Overall, though there were not many significant findings in this paper, the researchers presented some possible diet-dependent effects of Se on the microbiota that should spark further research.

Research in swine has also uncovered some significant findings in terms of the ability of Se to modulate the gut microbiota and host immune system [53]. One previously cited study, for example, found interesting differences in the composition of the intestinal microbiota of gilts that were fed diets either with 0.3 mg of sodium selenite/kg of feed, 0.3 mg of HMSeBA/kg of feed, or very low Se levels. At the phylum level, the HMSeBA-fed gilts had a significantly greater abundance of *Firmicutes* with fewer *Bacteroides*, *Melainabacteria*, and *Spirobacteria*. Interestingly, the researchers found that the serum IL-6 concentrations were positively correlated with the abundance of *Melainabacteria*, whereas *Firmicutes* were negatively correlated to the IL-6 levels. Overall, this study provided evidence that the modulation of these microbial species by dietary Se can impact the baseline levels of inflammatory cytokines, which may influence immune responses.

In chickens, the addition of dietary Se yeast has also yielded promising results in terms of microbiome and immune system modulation. In the previously mentioned study by Liu [55], several notable shifts in the microbiota of laying hens that received different dietary Se treatments for 12 weeks compared to the Se-deficient control group were linked to observed immunological effects. For example, one bacterial genus enhanced by Se yeast was *Veillonella*, which can produce immunomodulatory SCFAs [83]. On the other hand, *Stenotrophomonas* abundance was decreased by Se yeast. Since *Stenotrophomonas* is an opportunistic pathogen, this suggests that Se may be beneficial in reducing disease incidence. Moreover, the researcher’s further analyses found a correlation between the effects of Se yeast supplementation on the microbiota and ileal *IFNG* transcription. In general, the authors postulated that using Se to balance intestinal bacteria populations to favor beneficial genera may be one way of improving the host’s immune response.

Finally, studies using human participants have also shown that Se can modulate gut microbiota. One key Chinese study characterized the intestinal microbiota of seniors who lived in regions with high or low soil Se, where Se exposure was confirmed by blood and nail Se analysis [86]. No significant differences in the phenotypic classifications of the gut microbiota were observed in seniors from the high- or low-Se areas. However, these researchers found many significant variations in the activities of the gut microflora when comparing the two groups. Some notably different metabolic pathways between the two groups included DNA repair and recombination, cysteine and methionine metabolism, amino acid synthesis, and glyoxylate and dicarboxylate metabolism. Though the researchers did not directly connect these findings to immune system function, this study still demonstrated that the level of dietary Se is correlated with functions of intestinal microbial populations. Additionally, a recent randomized controlled trial looked at how the consumption of cashews and Brazil nuts, both rich in Se, impacted the microbiomes of overweight women [87]. In this study, women were energy-restricted (−500 kcal/day from their estimated energy requirement) for 8 weeks and were allocated into either the control group receiving no nuts or the mixed nuts group receiving 30 g of cashew nuts and 15 g of Brazil nuts daily. By the end of the study period, the women in the mixed nuts group had significantly increased abundances of *Ruminococcus*, *Roseburia*, and certain strains of *Ruminococceae* bacteria in their fecal samples compared with the control group. These bacteria are linked to positive health effects such as the production of propionate, an SCFA linked to an increase in tight junction proteins, antioxidant enzyme activity, and anti-inflammatory processes. However, some contentious bacteria also increased in the fecal samples of the women in the mixed nuts group, including those in the *Lachnoclostridium* genus, which is linked to obesity and high-lipid diets. Still, overall, the changes in the microbiome’s composition appeared to be positive according to the authors. Though Brazil nuts and cashews contain other beneficial compounds, such as unsaturated fatty acids, polyphenols, monounsaturated fatty acids, fiber, and omega-3 fatty acids, the researchers rightly or wrongly speculated that Se was a major contributing factor to the observed health improvements due to its antioxidant activities and the significant concomitant increase in plasma Se levels between the baseline and endpoint measurements in the mixed nuts group [87].

Overall, the current consensus in the scientific literature is that Se has an immunologically beneficial role in human and animal health, especially regarding its microbiota-modulating activity. Though many of the cited studies used animal models to test the efficacy of Se in modulating the microbiome, this study gives a solid foundation upon which to conduct more research with human participants. For example, future research using different forms of Se, dosing regimens, and time lengths of supplementation would be beneficial to optimize and isolate Se’s impact on the microbiome.

### 5.5. Disease Resistance

#### 5.5.1. Viral Infections

Researchers have well established the ability of viruses to elicit host ROS production by increasing the transcription of ROS-producing enzymes and reducing antioxidant processes [50]. Enhanced ROS production occurs in response to many viral infections, including hepatitis C, human immunodeficiency virus, hepatitis B, Epstein–Barr virus, herpes simplex virus type 1, respiratory syncytial virus, human T cell leukemia virus type 1, vesicular stomatitis virus, and influenza viruses [50]. As evidence of this, patients with chronic hepatitis B had significantly lower *GPX4* expression in their peripheral blood mononuclear cells in comparison to healthy controls, which was significantly and positively correlated with ROS and MDA levels [88]. When this increase in ROS production goes unchecked, it can put selection pressure on certain viruses to mutate and become more pathogenic [50] in addition to damaging host tissues. Se’s antioxidant function, as evidenced in GPxs and TrxRs, is therefore critical to restore these imbalanced intracellular redox states to homeostasis during viral infections.

Another selenoprotein that contributes to combatting viral infections is SelK. In West Nile virus-challenged mice, for example, researchers noted that *SELENOK* knockout mice had reduced serum IL-6 and MCP-1 concentrations in comparison to wild type mice [20]. However, the *SELENOK* knockout mice had higher levels of chemokine MCP-1 and inflammation in the brain compared to the wild type mice. These researchers hypothesized that the *SELENOK* knockout mice had poorer immune system activation and viral clearance, thus resulting in viral accumulation in the brain and neuroinflammation. In support of this hypothesis, they also observed higher West Nile virus titers in the brains and the sera of the *SELENOK* knockout mice in comparison to the wild type mice. Moreover, the survival rate of the wild type mice was 38.5% compared to 8.7% for the *SELENOK* knockout mice [20]. Overall, the *SELENOK* knockout mice displayed an impaired immune response to West Nile virus, highlighting the need for sufficient Se intake for adequate SelK synthesis.

A recent systematic review and meta-analysis also demonstrated that patients infected with severe acute respiratory syndrome coronavirus 2 (SARS-CoV-2) had lower serum Se levels compared to healthy individuals [89]. These authors postulated several potential conditions that could contribute to the low Se status during COVID-19 infection, including a potential negative effect of pharmaceutical treatments on Se absorption and excretion, attenuated renal function leading to higher Se excretion, an increased demand for Se resulting from the induction of stress pathways, or increased oxidative stress. It is also possible that low Se levels before infection increased the risk for these individuals to contract SARS-CoV-2 infection. Other authors performing a systematic review considered this after observing similar trends in lowered Se statuses and severe COVID-19 outcomes in the literature [90]. Overall, more research is needed to confirm and further explore the association between low Se levels and severe COVID-19 infection because administering Se supplementation to patients suffering from viruses like SARS-CoV-2 may help prevent severe disease and hasten convalescence.

#### 5.5.2. Bacterial Infections

Since Se deficiency is also linked to several bacterial infections, Se supplementation may assist in improving patient health outcomes [76]. As evidence of this, administering Se to animals infected with *E. coli*, *Clostridium perfringens*, *Listeria monocytogenes*, and other bacterial pathogens has proven valuable for recovery [76]. However, in some instances, supplementation with Se may not be advised, as certain pathogenic bacteria use Se to enhance their ability to invade host tissues. The pathogenicity of *Campylobacter jejuni*, for example, increases due to the expression of the selenoprotein gene *formate dehydrogenase A* [76]. *C. sticklandii* and *Enterococcus faecalis* infectivity may also be elevated by Se [76]. Though more research is needed in this area, conventional thinking is that the advantages of Se in ameliorating the host immune response outweigh the risks of increased bacterial pathogenicity. As this topic was reviewed in 2019 [76], only recent studies will be reviewed in detail below to update our understanding of how Se impacts globally relevant bacterial pathogens.

Tuberculosis caused by *Mycobacterium tuberculosis* is one fatal bacterial disease linked to Se deficiency. Consequently, the antioxidant and anti-inflammatory activities of orally administered Se are thought to improve patient recovery [91]. A variety of other therapeutic Se applications that can improve a patient’s resistance against this bacterial infection have also been explored. For example, menadione-derived selenoesters have been successfully developed to inhibit *M. tuberculosis* activity and evade its drug resistance [92]. Moreover, newly developed Se nanoparticles have been proven to dose-dependently inhibit the growth of *M. tuberculosis*, and they are less toxic than conventional organic and inorganic forms of Se [93]. These Se nanoparticles aid in destroying bacteria through the production of ROS, disturbing membrane potential, or damaging membrane integrity [93]. Moreover, in cases when a patient is co-infected with *M. tuberculosis* and the protozoan *Toxoplasma gondii*, Se nanoparticles may also be a successful treatment [94]. In support of this, Se nanoparticles have already shown immunostimulatory capabilities in mice infected with *T. gondii* by elevating the expression of *IL12*, *IFNG*, *TNFα*, and inducible nitric oxide synthase (iNOS), all of which are required to mount an effective antiparasitic immune response [94]. Thus, the use of Se nanoparticles in cases of *M. tuberculosis* and *T. gondii* co-infection may be promising.

*Staphylococcus aureus* is a Gram-positive bacterial pathogen that causes a significant burden on the global healthcare system due, in part, to its antibiotic resistance. As a treatment option, Se nanoparticles have also been extensively studied due to their bactericidal activity [95,96]. These Se nanoparticles assist with causing protein degradation as part of their antimicrobial effects [96]. Moreover, an *in vitro* study showed that when macrophages were infected with *S. aureus*, the addition of sodium selenite significantly attenuated the phosphorylation of key MAPK signaling pathway proteins such as Erk, p38, and JNK in comparison to infected macrophages not supplemented with Se [97]. Likewise, sodium selenite administration attenuated the phosphorylation of NF-κB proteins IκBα and p65. Since both pathways are involved in inflammatory responses, Se may be beneficial for eliciting anti-inflammatory activity during *S. aureus* infections to reduce systemic inflammation. Se also decreased the expression of autophagic protein p62 while increasing light chain 3 II expression. This modulation of protein expression contributes to the enhanced efficiency of autophagy, a catabolic mechanism that helps to combat infections through lysosomal degradation [97]. These authors concluded that Se elicits pro-autophagic and anti-inflammatory activities on macrophages *in vitro*, thus highlighting its efficacy in promoting the immune response against *S. aureus* and reducing its proliferation in macrophages. Other researchers confirm Se’s advantageous mechanisms of action in treating methicillin-resistant *S. aureus* by mobilizing leukocyte activation and promoting tissue repair [98]. This study was also performed using Se nanoparticles, showing their value as antibacterial agents.

Systemic bacterial infections are also causative agents of sepsis, a costly global healthcare problem. Sepsis can result from excessive inflammation and oxidative stress, and it can lead to unnecessary coagulation and multi-organ dysfunction that can result in death [99]. A link between the administration of Se to septic patients and reduced all-cause mortality and duration of hospital stay was recently discovered in a meta-analysis of randomized controlled trials [99]. Though Se administration did not significantly affect mortality after 28 days, the rate of acute renal failure, length of vasopressor therapy, or other adverse health events, it may still be a worthwhile therapy due to its relative safety and beneficial immunomodulatory effects [99]. The development of novel porous Se nanozymes is also being considered to treat sepsis [100]. In a lipopolysaccharide (LPS) endotoxin model of sepsis [100], Chen et al. found that the serum concentrations of pro-inflammatory cytokines TNF-α, IL-6, and IL-1β were decreased in mice in the LPS-treated groups that received Se nanozymes in comparison to the positive control group. Additionally, biomarkers for lung and liver degeneration were reduced to normal levels after 15 days of treatment with the mesoporous Se–hyaluronic acid nanozyme. Leukocyte infiltration, hemorrhaging, and ROS production were also decreased with the Se nanozyme treatments compared with the positive control group. Finally, the death rate of LPS-challenged mice declined significantly with the Se nanozyme treatment. Therefore, applying this novel nanotechnology to the medical world may be incredibly valuable for treating sepsis and other ROS-generating inflammatory disorders. Further *in vivo* studies to test the efficacy and safety of these Se-based nanotechnology treatments in comparison to traditional approaches should be carried out.

#### 5.5.3. Fungal Infections

Fungi are another important disease-causing agent affecting humans that Se may help to combat [101]. Two pathogenic fungi include the yeast of the *Candida* genus and the filamentous fungi of the *Fusarium* genus [102]. One way that fungi, like *Fusarium*, elicit their toxic effects is through mycotoxins, such as deoxynivalenol, zearalenone, and fumonisins [102]. However, nano-Se synthesized by microorganisms from human breast milk has demonstrated antifungal activity by inhibiting the growth of different species of *Candida* and *Fusarium* through its penetration and destruction of cellular structures [88]. Many other studies have confirmed the fungicidal and fungistatic properties of Se nanoparticles against other harmful fungi, including *Aspergillus niger*, *Colletotrichum capsici*, and *Candida albicans* [101,103,104]. A few antifungal mechanisms of action through which nano-Se is thought to act include destroying cellular walls, inactivating enzymes, protein degradation, reducing ATP production, enhancing ROS production, and impairing cellular integrity [101]. Moreover, since nano-Se can be biosynthesized in a safe, efficient, and economical way by microorganisms, this may be an environmentally friendly way to produce viable nano-Se treatments in comparison to conventional chemical and physical processes used to make nano-Se [105]. Therefore, as fungi become increasingly resistant to antifungal pharmaceutical treatments, alternative, non-toxic options must be pursued, which is why nano-Se should be researched further as a potential treatment option [101]. The safety of these biosynthesized nano-Se particles in humans and potential mechanisms for toxicity should also be evaluated before their implementation into clinical practice.

#### 5.5.4. Autoimmune Disorders

The most notable autoimmune disorder that may be linked to Se deficiency is autoimmune thyroiditis, which occurs when autoantibodies attack the thyroid tissue, leading to goiters, dysphagia, neck compression, and pharyngeal pain [106]. Thyroid peroxidase antibodies and thyroglobulin antibodies generated by differentiated B cells contribute to thyroid cell damage [106]. While Se’s antioxidant function plays a role in autoimmune thyroiditis prevention, its modulation of adaptive immune responses may also be useful for prophylaxis [106]. One 2021 prospective randomized controlled trial tested the therapeutic effect of 200 µg Se yeast tablets administered daily for six months to patients with Hashimoto’s thyroiditis [107]. These researchers found that Se-supplemented patients had higher percentages of activated regulatory T cells (Tregs) compared to the non-supplemented control group [107]. These Tregs are essential for promoting immune system homeostasis and self-tolerance; thus, increasing Tregs is beneficial for ameliorating autoimmune disorders like Hashimoto’s thyroiditis. Additionally, this study found that Se supplementation significantly reduced serum levels of thyroid peroxidase and thyroglobulin antibodies after six months, illustrating Se’s ability to attenuate the production of destructive autoantibodies. Finally, the serum GPx3 concentrations also increased in the Se-supplemented group in comparison to the control group, which shows that it may have been beneficial for treating these patients due to its antioxidant activity. Therefore, this study demonstrated promising results to support the administration of Se to patients with autoimmune thyroiditis. A 2023 systemic review and meta-analysis on the possible benefits of Se on Hashimoto thyroiditis provided conservative evidence for supplementing patients with Hashimoto thyroiditis with Se over 6 months by demonstrating an associated decrease in thyroid peroxidase and thyroglobulin antibodies [108]. Moreover, a recent study found an association between low plasma Se concentrations and markers for thyroid autoimmunity and preeclampsia in pregnant women, highlighting the importance of having an adequate Se status during the gestational period as well to prevent adverse health events [109]. However, a 2023 systemic review on the therapeutic impact of Se supplementation on autoimmune thyroiditis found a low certainty of evidence to support applying Se supplementation to clinical practice [106]. Though Se supplementation lowered thyroid peroxidase and thyroglobulin antibodies at the 3- and 6-month study marks, further research is needed to confirm these results over a longer period. While Se supplementation may appear advantageous for patients with autoimmune thyroiditis, researchers must conduct more long-term studies and focus on how Se supplementation affects thyroid function before its implementation at the clinical level [108].

Ulcerative colitis is another autoimmune disorder that may be ameliorated by Se supplementation [110]. In one randomized double-blind placebo-controlled clinical trial, patients were supplemented with 200 µg of selenomethionine per day over ten weeks [111]. In comparison to the non-supplemented control group, the concentration of serum inflammatory cytokine IL-17 decreased significantly in the patients receiving Se. IL-17 is pro-inflammatory cytokine secreted by T cells that enhances the production of other inflammatory mediators and is implicated in the pathogenesis of ulcerative colitis [112]. Additionally, the supplemented patients reported an improved quality of life and a lower score on the simple clinical colitis activity index by the end of the trial compared to the non-supplemented groups, revealing Se’s tangible value in treating disease symptoms. Moreover, a recent case–control study provided evidence that ulcerative patients with flare-ups had significantly lower serum Se concentrations compared to healthy individuals or those in remission, signifying that Se may play a role in the pathogenesis of this condition [113]. Overall, implementing Se supplementation in treatment protocols for patients with ulcerative colitis and other inflammatory autoimmune gastrointestinal disorders may offer various benefits due to Se’s microbiome-modulating, immunomodulating, anti-inflammatory, and antioxidant activities [110]. Thus, further research should be completed to verify its therapeutic potential and dosing guidelines over longer time periods.

#### 5.5.5. Cancer

Immunotherapy, which harnesses the immune system to fight against cancerous cells, is a promising approach for treating various cancers [114], and Se can be employed to achieve this goal. For example, Se can increase NK cell activation and reduce the fiber protein structure covering tumor cells to promote better antigen access by NK cells [62]. Se’s reduction in ferroptosis is also noted as one of its anti-cancer properties [115]. Additionally, Se can act in a pro-oxidant and pro-inflammatory way to help induce cancer cell death [116]. In a similar way, Se nanoparticles can directly regulate the activities of cells of the innate and the adaptive immune system. For example, Se nanoparticles can direct macrophage immune responses against cancer cells [114]. Specifically, in a recent paper, researchers successfully used *Pholiota adiposa* polysaccharide-coated Se nanoparticles to cause hepatoma cell death *in vitro* by polarizing M2 macrophages to the M1 phenotype and enhancing cytokine production [117]. Moreover, this nanoparticle treatment also elevated the number of CD8^+^ and CD4^+^ T cells to enhance the anti-tumor immune response.

When assessing the safety of these nanoparticles *in vivo*, the researchers found that the treated mice showed no adverse effects [117]. An alternative application of Se nanoparticles has been in conjunction with manganese in a biosynthetic MnSe nanobomb, which proved to effectively induce ROS production and the anti-tumor immune response through the cGAS-STING signaling pathways, causing leukocytes to infiltrate the tumor site [118]. Otherwise, Se nanoparticles can also act as antioxidant factors, drug carriers, and anti-inflammatory agents, all of which can benefit patients with cancer, making Se nanoparticles a favorable therapeutic option [119].

One interesting aspect of Se nanoparticles is their cytotoxic effects, which may be used for cancer therapeutics. A research group studied the effects of these particles on glioblastoma, colorectal adenocarcinoma, prostate carcinoma, and breast adenocarcinoma cancer cell lines [120]. Though the cell lines were not all equally affected, the Se nanoparticles generally upregulated the expression of pro-apoptotic genes *DDIT3*, *PPP1R15A*, *BCL2L11*, and *BBC3*, as well as the endoplasmic reticulum stress response. Se nanoparticles also decreased cancer cell viability and proliferation while increasing the mRNA expression of *TXN*, *GPX1*, *GPX2*, *GPX3*, and *GPX4* in many of the cancer cell lines. One potential contributor to the pro-apoptotic and anti-proliferative effect was the increase in Ca^2+^ signaling induced by the Se nanoparticles. Importantly, Se did not have any toxic effects on healthy control mouse fibroblast cells, showing potential selectivity in its cytotoxic effects against cancer cells. Moreover, the Se nanoparticles in the aforementioned study also inhibited tumor cell proliferation while promoting apoptosis [117]. Overall, these studies provide hopeful evidence for the future application of Se nanoparticles in anti-tumor cancer therapy.

Not all studies, however, have shown that Se influences cancer incidence. In a randomized trial, patients in one of the treatment groups received 200 µg of L-selenomethione per day over 4.6 years, but no significant impact on adenomas in the colon or the rectum was observed [121]. This points to the possibility that different forms of Se or routes of administration may be more efficient for cancer therapies. Therefore, Se has proven to be beneficial for some anti-cancer applications, and the development of novel Se nanoparticles warrants further investigation.

## 6. Conclusions

Se is a versatile micronutrient required for the function of all cells, including leukocytes of the immune system. Without sufficient Se, inflammation and ROS/RNS production can go unchecked. Suboptimal cellular signaling and pathogen clearance can also occur. Therefore, as Se journeys from the environment to the body through various routes, it is clearly a mineral we cannot do without. Whether found in dietary sources, supplements, or nanoparticles, Se is necessary to support all aspects of optimal immune function and a person’s resistance to disease, as summarized in Table 2.

However, more research is needed to establish optimal therapeutic protocols, including ways to monitor Se levels, to ensure immunological efficacy without toxicity. Scientific questions that remain to be explored include the following: Which form of Se (organic, inorganic, or nanoparticle sources) is the most appropriate for nutritional or therapeutic interventions? Should daily Se requirements or therapeutic supplementation rates take into consideration body weight and the form of Se administered? Can Se nanoparticles be safely administered in humans with drug-resistant microbes? Can supranutritional doses of Se be routinely administered to patients with certain diseases without adverse effects? How much Se is too much? How can we best take advantage of Se’s ROS generating effects and ROS scavenging effects to improve the outcomes of patients with cancer? All these questions are worth asking due to the essentiality of immunomodulatory research in giving doctors and medical workers the necessary tools to promote the health of our growing global population in a world with rising challenges such as novel pathogens, antibiotic resistance, obesity, cancers, and autoimmune disorders.

## Figures and Tables

**Table 1:** Selenium requirements by age [23]. EARs: Estimated Average Requirements; RDAs: Recommended Dietary Allowances; UL: Tolerable Upper Limits.

**Infants**			
**Age**	EARs (µg/Day)	RDAs (µg/Day)	ULs (µg/Day)
**0–6 months**	No data	15	45
**7–12 months**	No data	20	60
**Children**			
**Age**	EAR (µg/day)	RDA (µg/day)	UL (µg/day)
**1–3 years**	17	20	90
**4–8 years**	23	30	150
**Men**			
**Age**	EAR (µg/day)	RDA (µg/day)	UL (µg/day)
**9–13 years**	35	40	280
**14–>70 years**	45	55	400
**Women**			
**Age**	EAR (µg/day)	RDA (µg/day)	UL (µg/day)
**9–13 years**	35	40	280
**14–>70 years**	45	55	400
**Pregnant Women**			
**Age**	EAR (µg/day)	RDA (µg/day)	UL (µg/day)
**<18–50 years**	49	60	400
**Lactating Women**			
**Age**	EAR (µg/day)	RDA (µg/day)	UL (µg/day)
**<18–50 years**	59	70	400

**Table 2:** The benefits of therapeutic and nutritional interventions using Se.

Selenium Interventions	Potential Benefits	Conditions Targeted
Dietary Selenium	Maintenance of human health and normal physiological processes [40]	Prevention of disorders linked to Se deficiency, including Kashin–Beck and Keshan disease [40]
Supranutritional Oral Supplementation of Selenium	Dose dependent antioxidant activities [37,53,56] Reduction in inflammation and oxidative damage [53] Enhanced antibody production and immune responses against pathogens [53,56,70] Reduced auto-antibody production [107]	Inflammatory Conditions: Reduction in biomarkers associated with inflammation in Alzheimer’s disease [63] Bacterial Infections: Immunostimulatory effects against pathogenic bacteria and improved health outcomes [56,72,76,91] Viral Infections: Lowered mutation rate of viruses during infections [50] Improved health outcomes during infection [20,89,90] Microbiome Resilience: Increased abundance of beneficial bacteria [77,83,87] Improved gut barrier function [77] Enhanced SCFA production [77] Reduced inflammation [53,77] Autoimmune Disorders: Improved health parameters in those with autoimmune disorders, such as autoimmune thyroiditis and ulcerative colitis [107,109,110,113]
Selenium-Based Nanotechnologies	Superior bioavailability [31] Enhanced antioxidant, anti-inflammatory, and immunomodulatory activities [56,57,100] Targeted toxic mechanisms of action against cancer cells and pathogens without harming healthy cells [31,117,120]	Bacterial, Fungal, and Protozoan Infections: Reduced pathogenicity [88,92,93,94] and improved health outcomes [100] Cancer: Immunotherapy against cancer cells [114,117,118,119,120]
